# Measuring self-efficacy in patient-centeredness of physicians in oncology – translation, adaptation and evaluation of psychometric properties of the SEPCQ-27 German version (SEPCQ-24-GER)

**DOI:** 10.1080/07853890.2025.2468255

**Published:** 2025-03-03

**Authors:** Christian Heuser, Nicole Ernstmann, André Karger, Andrea Petermann-Meyer, Franziska Geiser, Frank Vitinius, Luisa Ernsten, Robert Zachariae, Kati Hiltrop

**Affiliations:** a Chair of Health Services Research, Institute of Medical Sociology, Health Services Research, and Rehabilitation Science (IMVR), Faculty of Medicine and University Hospital Cologne, University of Cologne, Cologne, Germany; b Center for Health Communication and Health Services Research (CHSR), Department of Psychosomatic Medicine and Psychotherapy, University Hospital Bonn, Bonn, Germany; c Center for Integrated Oncology Aachen, Bonn, Cologne, Düsseldorf (CIO ABCD), Cologne, Germany; d Clinical Institute of Psychosomatic Medicine and Psychotherapy, University Hospital Düsseldorf, Medical Faculty, Heinrich-Heine University Düsseldorf, Düsseldorf, Germany; e Department of Hematology, Oncology, Hemostaseology and Stem Cell Transplantation, Faculty of Medicine, RWTH Aachen University, Aachen, Germany; f Department of Psychosomatic Medicine and Psychotherapy, University Hospital Bonn, Bonn, Germany; g Department of Psychosomatics and Psychotherapy, University of Cologne, Faculty of Medicine and University Hospital Cologne, Cologne, Germany; h Department of Psychosomatic Medicine, Robert-Bosch-Hospital, Stuttgart, Germany; i Unit for Psychooncology and Health Psychology, Department of Oncology, Aarhus University Hospital and Department of Psychology, Aarhus University, Denmark

**Keywords:** Patient-centered communication, patient-centeredness, self-efficacy, validation, psychometric properties, physicians, oncology, health services research, SEPCQ-27, SEPCQ-24-GER

## Abstract

**Introduction:**

Providers’ self-efficacy in patient-centeredness, defined as their confidence in their ability to communicate in a patient-centered manner, is linked to their patient-centered attitudes and behaviors. The SEPCQ-27 is a validated instrument to measure medical students’ and physicians’ self-efficacy in patient-centered communication. The aim of this study was to produce a German adaptation of the SEPCQ-27 and evaluate its psychometric properties in a sample of physicians in oncology.

**Materials and methods:**

The SEPCQ-27 was professionally translated and adapted into German. Descriptive analyses, exploratory and confirmatory factor analyses, and internal consistency, convergent, discriminant and construct validity were conducted using data from a sample of *n* = 258 physicians collected during the three-arm cluster-randomized intervention trial ‘KommRhein Interpro’ conducted from 2019–2023.

**Results:**

Exploratory factor analysis led to a modified three-dimensional German version with 24 items (SEPCQ-24-GER), which showed acceptable fit in confirmatory factor analysis (*χ^2^/df* = 1.8, CFI = 0.92, TLI = 0.91, SRMR = 0.05, RMSEA = 0.06). The SEPCQ-24-GER demonstrated good internal consistency (Cronbach’s alpha > 0.7 for all three dimensions) and acceptable convergent (CR for all dimensions is > 0.7), discriminant (√AVE is higher than the factor intercorrelations for all but one factor), and construct validity (regarding occupational group (*F* = 4.741, *p* < .001), occupational experience (*r* =.240, *p* < .001) and between factor 3 and sex (*t* = 2.575, *p* = .011)).

**Discussion:**

The SEPCQ-24-GER demonstrated satisfactory psychometric properties. Future research should focus on further measures of reliability, sensitivity to change and validation within different samples.

**Trial Registration:**

DRKS (German Clinical Trials Register) - ID: DRKS00022563, registered 29/07/2020.

## Introduction

Patient-centered communication in oncology is a key element of high-quality treatment and care and a core competence of physicians and other healthcare providers (HCP) in modern healthcare [1–3]. The core aspects of patient centeredness, which is the conceptual framework of this study [4] and defined as medical and psychosocial care that is oriented towards the individual preferences, needs and values of patients [1], include HCP’s empathic and trustful communication with patients aimed at understanding patients’ individual psychosocial context and promoting preference-based shared involvement of patients in treatment planning and decision-making over the course of their treatment [1,2,5,6]. In oncology, patient-centered communication has been found associated with positive patient treatment outcomes such as treatment satisfaction, adherence, and (more controversially) health status [7–11]. In oncology, patient-centered communication behaviors have been reported to be associated with markers of physicians’ subjective well-being, including levels of stress, rate of burnout, job satisfaction, and the level of empathy in their interactions with patients [12–15].

Communication skills are thus a core component in medical educations internationally and also in Germany. Physicians’ communication skills can be improved by communication skills training in oncology with positive effects on cancer patients’ outcomes, e.g. patient satisfaction and patient distress [16–21]. In the German context, existing communication skills training courses appear to strengthen personal learning achievements, self-efficacy, communicative competencies, and successful practice transfer [22], with a preference for short training courses that are compatible with clinical practice [23]. Most communication trainings are skill-oriented and aim to improve concrete communicative skills in prototypical conversational situations [24]. Physicians’ patient communication skills and related competencies can be measured with different methods. One approach is observer-based ratings, but this approach is time-consuming [25]. Another is using questionnaire-based assessment [24,26,27].

In the acquisition of knowledge, attitudes, and skills, increasing attention is being paid to the role that physicians’ beliefs in their own competencies play in this process [28]. This aspect is the focus of SEPCQ-27, a recently developed questionnaire which assesses physicians’ self-efficacy in patient-centeredness [25]. The instrument is based on Bandura’s concept of self-efficacy, which is defined as ‘beliefs in one’s capabilities to organize and execute the courses of action required to produce given attainments’ [29]. In SEPCQ-27, respondents are thus asked to report on their confidence in their ability to exert a number of specific skills and behaviors associated with the concept of patient-centeredness in a manner so that the patient would perceive it according to the underlying intention [25]. Research has confirmed that providers’ self-efficacy in patient-centeredness, as ‘the confidence to perform actions on the basis of their communication skills’ [30], is linked to exhibiting more patient-centered behaviors and attitudes [31–33]. So far, the SEPCQ-27 has been used to measure physicians’ and medical students’ self-efficacy in patient-centered communication [25,34]. The questionnaire is available in Danish and English, however, no German version is available yet. The aim of the present study was therefore to translate and adapt the SEPCQ-27 to the German context and to evaluate its psychometric properties with physicians in oncology.

## Materials and methods

The methods section is based on the COSMIN guideline for assessing the methodological quality of studies on measurement properties [35]. A classical test theory approach (rather than item response theory) was followed in order to be comparable to the original SEPCQ-27 version [25].

### Study design

This study was part of the larger three-arm cluster-randomized intervention trial ‘KommRhein Interpro’ (Effectiveness of an Interprofessional Communication Skills Training for Oncology Teams) conducted at the Cancer Centers of the University Hospitals of Aachen, Bonn, Cologne and Düsseldorf (CIO ABCD) between July 2019 and June 2023. Further information on the study design can be found elsewhere [36].

### Sample and data collection

Study nurses screened the physicians on the 30 participating oncology wards for eligibility with the help of a standardized screening document. Inclusion criteria were a physician’s medical license to practice, an assignment to a participating ward unit in the Cancer Centers, age ≥ 18 years, sufficient German language skills, and written informed consent. Due to high turnover and a rotation system between the different ward units, it was not possible to perform a power calculation. Instead, the number of physicians prior to data collection was calculated for each ward unit, resulting in an estimated range of 150–180 physicians (5–6 per ward). Finally, data from 258 physicians were collected. Data from T0 were used for this study. Data collection was conducted according to the Total Design Method by Dillman with two postal reminders [37].

### Measure

In general, the questionnaires for physicians consisted of several measures which are described elsewhere [36]. Here sociodemographics (self-developed; including sex, age, occupation, work experience) [36] and the Self-Efficacy in Patient-Centeredness Questionnaire (SEPCQ-27) was used. The SEPCQ-27 consists of 27 items belonging to three factors. A 5-point Likert scale (0 = ‘to a very low degree’ to 4 = ‘to a very high degree’) is used. The three factors are: (1) exploring the patient perspective, (2) sharing information and power, (3) dealing with communicative challenges. The score ranges from 27–135 with previously demonstrated mean scores of 64.2–70.1 in medical students and physicians [25]. The internal consistencies (Cronbach’s *α*) ranged between 0.92 and 0.95 [25]. The SEPCQ-27 was adapted from English into German following the procedures recommended by Guillemin et al. [38]. First, three professional translators independently translated the English questionnaire into German. Three researchers outside the study team appraised the three German versions and the most appropriate version for each item was chosen leading to a preliminary German version. This preliminary German version was discussed in a consensus meeting of the whole study team (physicians, nurses, psychologists and sociologists). The agreed upon German version was then back-translated into English independently by three professional translators. The three back-translated versions were again appraised by three researchers outside the study team to identify the most similar version per item compared to the original wording. The final back-translation was discussed in the study team (CH, NE, AK, LE, KH) and sent to the first author of the original SEPCQ-27 version (RZ) for approval. Three pilot pretests were performed with physicians and nurses using the ‘thinking aloud method’ [39] resulting in adaptations and linguistic simplifications, e.g. concerning the introduction text of scales and the whole questionnaire.

### Data analysis

IBM SPSS Version 27 (descriptive analyses, exploratory factor analysis, Cronbach’s *α*) and AMOS Version 27 (confirmatory factor analysis) were used for the analyses. Of the 258 physicians, 10 (2.6%) had missing values on single items. Missing values ranged from 0% to 1.2% per single item. These cases were imputed with the help of the expectation-maximization-algorithm (EM-algorithm) [40] leading to *n* = 258 included cases for the analysis.

### Descriptive analyses

Descriptive analyses of the sample characteristics (mean, median, minimum-maximum, standard deviation) and the 27 and 24 items (means, standard deviations, medians, skewness, minimum, maximum) were performed.

### Exploratory factor analysis (EFA)

To evaluate the suitability of the data for EFA, the Kaiser-Meyer-Olkin (KMO > 0.5 mediocre) and measure of sampling adequacy (MSA > 0.5) were used [41]. The appropriateness for EFA was evaluated with the help of a significant Bartlett’s test (< 0.05) indicating that the correlations between items were significantly different from zero [41]. Principal component analysis with orthogonal Varimax rotation was performed. The number of extracted factors was guided by Kaiser’s criterion (eigenvalues > 1) and the screeplot [41]. Factor loadings > 0.4 would be evaluated as significant and cross-loadings < 0.4 as acceptable [42].

### Confirmatory factor analysis (CFA)

The following overall indices were used in accordance with the current literature [42]: normed *χ^2^* (*χ^2^/df* ≤ 2 good) with a significant *p*-value, comparative fit index (CFI ≥ 0.92), Tucker-Lewis Index (TLI ≥ 0.92), standardized root mean square residual (SRMR < 0.08) and root mean square error of approximation (RMSEA < 0.07).

### Internal consistency, convergent, discriminant and construct validity

Internal consistencies (Cronbach’s *α*; > 0.7 acceptable) were calculated for the resulting SEPCQ-24-GER total and its three subscales [41,42]. To evaluate convergent validity of a dimension, the average extracted variance (AVE ≥ 0.5) and composite reliability (CR > 0.7 good) were used [42]. The root square of the AVE (√AVE) was used as an indicator for discriminant validity and, following the ‘Fornell Larcker criterion’, expected to be higher than the correlations with other dimensions [43]. In terms of construct validity, which means that one construct is related to another construct at some level, the SEPCQ-24-GER factor mean subscores and overall mean score were analyzed with sex (*t*-test), occupational group (ANOVA), and occupational experience (correlation), with the hypotheses that men (medium effect), higher hierarchy levels (strong effect) and more years of occupational experience (strong effect) would score higher on self-efficacy in general and therefore on the SEPCQ-24-GER in particular.

## Results

### Descriptive analyses

The characteristics of the total sample of 258 physicians are shown in Table 1. The mean age was 35.5 (median 33) years overall, 33.4 (median 31) years for women, and 37.8 (median 36) years for men. Sex was equally distributed. The average occupational experience was 8.6 years varying between sex with 6.7 (median 5) years for women and 10.4 (median 9) years for men. Half of the sample consisted of assistant physicians with fewer mean and median occupational experience.

**Table 1.** Sample characteristics.

Sex (*n*, (%))	Female	130 (50.4)
	Male	127 (49.2)
	Divers/Non-binary	1 (0.4)
Age (in years)	*n*	258
	Mean (SD)	35.5 (8.2)
	Median	33
Occupational group (*n*, (%))	Chief physician/Medical director	5 (1.9)
	Senior physician	78 (30.2)
	Ward physician	18 (7.0)
	Specialist physician	29 (11.2)
	Assistant physician	128 (49.6)
Occupational experience (in years)	*n*	258
	Mean (*SD*)	8.6 (8.1)
	Median	6

*Note*. Total *n* = 258.

Table 2 presents the descriptive statistics of the items and their corresponding dimensions in the original 27-items and the adapted 24-items model. The items were measured on a scale from 0 to 4 with means varying from 2.44 to 3.33 with median values from 2.00 to 3.00. Skewness of the items ranges from −1.177 to 0.052.

**Table 2.** Descriptive statistics of the SEPCQ-27 and SEPCQ-24-GER items and its corresponding dimensions in the original model.

Item no.	Dimension/Item	*M*	*SD*	*Md*	*S*	*Min*	*Max*
Exploring the patient perspective							
1.	Make the patient feel that I am genuinely interested in knowing what he/she thinks about his/her situation	3.17	0.662	3.00	−0.616	0	4
4.	Make the patient feel that I have time to listen	2.81	0.891	3.00	−0.588	0	4
5.	Recognize the patient’s thoughts and feelings	3.08	0.756	3.00	−0.482	1	4
9.	Be attentive and responsive	3.01	0.708	3.00	−0.218	1	4
10.	Be aware of when the patient is scared or concerned	3.25	0.703	3.00	−0.667	1	4
14.	Treat the patient in a caring manner	3.18	0.739	3.00	−0.727	0	4
17.	Make the patient experience me as empathic	3.19	0.709	3.00	−0.771	0	4
20.	Make the patient feel that he/she can talk with me about confidential, personal issues	3.10	0.752	3.00	−0.507	0	4
23.	Show a genuine interest in the patient and his/her situation	3.15	0.682	3.00	−0.654	0	4
24.*	Focus on compassion, care and symptomatic treatment, when there is no curative treatment	3.03	0.789	3.00	−0.606	1	4
Sharing information and power							
2.	Record a complete medical history	3.18	0.769	3.00	−1.068	0	4
6.	Reach agreement with the patient about the treatment plan to be implemented	3.21	0.693	3.00	−0.666	1	4
7.	Advise and support the patient in making decisions about his/her treatment	3.23	0.714	3.00	−0.839	1	4
11.	Ensure that the patient makes his/her decisions on an informed basis	3.10	0.747	3.00	−0.394	1	4
12.	Explain the diagnosis and treatment plan to the patient so that he/she understands	3.31	0.771	3.00	−1.177	0	4
15.	Explain things so that the patient feels well-informed	3.22	0.759	3.00	−1.004	0	4
18.	Inform the patient about the expected side effects, so the patient understands them	3.13	0.716	3.00	−0.535	1	4
21.	Explain how the treatment works or is expected to work	3.33	0.646	3.00	−0.724	1	4
25.	Explain how the treatment is likely to affect the patient’s condition, so that the patient understands	3.01	0.723	3.00	−0.408	1	4
26.	Explain the treatment procedures, so that the patient understands them	3.28	0.686	3.00	−0.885	0	4
Dealing with communicative challenges							
3.	Accept when there is no longer curative treatment for the patient	3.18	0.820	3.00	−0.916	0	4
8.	Be aware of when my own feelings affect my communication with the patient	2.72	0.840	3.00	−0.295	0	4
13.	Deal with my own emotional reactions when the situation is difficult for me	2.71	0.919	3.00	−0.347	0	4
16.*	Maintain the relationship with the patient when he/she is angry	2.44	0.946	3.00	−0.287	0	4
19.*	Stay focused on what is best for the patient if there is a professional disagreement about the diagnosis and treatment	2.91	0.739	3.00	−0.276	1	4
22.	Avoid letting myself be influenced by preconceptions about the patient	2.44	0.828	2.00	0.052	1	4
27.	Separate my personal views from my approach in the professional situation	3.85	0.815	3.00	−0.344	0	4
SEPCQ-27, factor 1, Exploring the patient perspective: *M* = 31.0, *SD* = 5.2, *Md* = 31; (in the original validation study mean was 32.4, *SD* = 4.5)							
SEPCQ-27, factor 2, Sharing information and power: *M* = 32.0, *SD* = 5.2, *Md* = 32; (in the original validation study mean was 31.9, *SD* = 4.9)							
SEPCQ-27, factor 3, Dealing with communicative challenges: *M* = 19.2, *SD* = 3.8, *Md* = 19; (in the original validation study mean was 19.7, SD = 3.7)							
SEPCQ-27 score: *M* = 82.3, *SD* = 12.5, *Md* = 82; (in the original validation study mean was 83.9, *SD* = 11.4)							
SEPCQ-24-GER, factor 1, Exploring the patient perspective: *M* = 27.9, *SD* = 4.7, *Md* = 28							
SEPCQ-24-GER, factor 2, Sharing information and power *M* = 32.0, *SD* = 5.2, *Md* = 32							
SEPCQ-24-GER, factor 3, Dealing with communicative challenges: *M* = 13.9, *SD* = 2.8, *Md* = 14							
SEPCQ-24-GER score: *M* = 73.8, *SD* = 11.1, *Md* = 74							

*Note. n* = 258; * items 16, 19 and 24 were omitted resulting in the SEPCQ-24-GER version.

### Exploratory factor analysis

KMO was 0.93, MSA ranged from 0.80 to 0.96, and Bartlett’s test was highly significant (*p* < .001), indicating suitability of the data for EFA. EFA resulted in a modified three-factor model with 27 items which is presented in Table 3. In this modified model, the items 16, 19 and 24 loaded on different factors compared to the original model.

**Table 3.** Modified EFA-based model with 27 items.

Modified EFA-based model		Rotated factor loadings	Differences compared to original model
Exploring the patient perspective			
1.	Make the patient feel that I am genuinely interested in knowing what he/she thinks about his/her situation	0.623	
4.	Make the patient feel that I have time to listen	0.529	
5.	Recognize the patient’s thoughts and feelings	0.686	
9.	Be attentive and responsive	0.609	
10.	Be aware of when the patient is scared or concerned	0.618	
14.	Treat the patient in a caring manner	0.655	
*16.*	*To maintain the relationship with the patient when he/she is angry*	*0.498*	*Item originally loads on 3rd dimension*
17.	Make the patient experience me as empathetic	0.743	
20.	Make the patient feel that he/she can talk with me about confidential, personal issues	0.672	
23.	Show a genuine interest in the patient and his/her situation	0.632	
Sharing information and power			
2.	Record a complete medical history	0.411	
6.	Reach agreement with the patient about the treatment plan to be implemented	0.579	
7.	Advise and support the patient in making decisions about his/her treatment	0.561	
11.	Ensure that the patient makes his/her decisions on an informed basis	0.652	
12.	Explain the diagnosis and treatment plan to the patient so that he/she understands	0.795	
15.	Explain things so that the patient feels well-informed	0.652	
18.	Inform the patient about the expected side effects, so the patient understands them	0.768	
*19.*	*To stay focused on what is best for the patient if there is a professional disagreement about the diagnosis and treatment*	*0.571*	*Item originally loads on 3rd dimension*
21.	Explain how the treatment works or is expected to work	0.721	
25.	Explain how the treatment is likely to affect the patient’s condition, so that the patient understands	0.620	
26.	Explain the treatment procedures, so that the patient understands them	0.655	
Dealing with communicative challenges			
3.	Accept when there is no longer curative treatment for the patient	0.412	
8.	Be aware of when my own feelings affect my communication with the patient	0.529	
13.	Deal with my own emotional reactions when the situation is difficult for me	0.750	
22.	Avoid letting myself be influenced by preconceptions about the patient	0.580	
*24.*	*Focus on compassion, care and symptomatic treatment, when there is no curative treatment*	*0.595*	*Item originally loads on 1st dimension*
27.	Separate my personal views from my approach in the professional situation	0.617	

*Note. n* = 258.

### Confirmatory factor analysis

In the EFA, the items 16, 19, and 24 loaded on different factors compared to the original model. Therefore, CFA was used to test the fit of the present data to (1) the original model of the SEPCQ-27, (2) the modified EFA-based model with 27 items and (3) the reduced modified EFA-based model (SEPCQ-24-GER) omitting the items 16, 19 and 24 which loaded on a different factor in EFA. Table 4 presents the models with their fit indices after allowing three error terms to correlate per model. The reduced EFA-based model with 24 items (SEPCQ-24-GER) resulted in satisfying fit indices, only just failing to reach the defined threshold of the TLI, and therefore present a better fitted model than the remaining two models.

**Table 4.** Fit indicees of different models.

	*χ²/df*	CFI	TLI	SRMR	RMSEA
Threshold	≤2.5	≥0.92	≥0.92	<0.08	<0.07
1) Original model	**2.0**	0.88	0.87	**0.06**	**0.06**
2) Modified EFA-based model with 27 items	**2.0**	0.89	0.87	**0.05**	**0.06**
3) Reduced modified EFA-based model with 24 items	**1.8**	**0.92**	0.91	**0.05**	**0.06**

*Note. n* = 258; values within the defined ranges for acceptable fit in bold; CFI: comparative fit index; TLI: Tucker-Lewis index; SRMR: standardized root mean square residual; RMSEA: root mean square error of approximation.

### Internal consistency, convergent, discriminant and construct validity

For the resulting SEPCQ-24-GER, Cronbach’s alphas > 0.7 were found for all three dimensions and for the overall score (0.93). Concerning convergent validity, the AVE failed to exceed the benchmark of 0.5 for all three dimensions, while the CR for all dimensions is > 0.7. For discriminant validity, the √AVE for all factors is higher than the factor intercorrelations except for factor 2–1 intercorrelation. Table 5 shows the internal consistency, validity measures and factor intercorrelations. Concerning construct validity, comparable variables to those in the original study [25] were selected. Significant differences between SEPCQ-24-GER factor mean subscores and overall mean score were found regarding occupational group (*F* = 4.741, *p* < .001) and occupational experience (*r* = .240, *p* < .001) as well as between factor 3 and sex (*t* = 2.575, *p* = .011). All effect sizes are low to moderate. Table 6 shows the detailed construct validity results.

**Table 5.** Internal consistency, convergent and discriminant validity of the SEPCQ-24-GER.

Dimension	Cronbach’s alpha	AVE	√AVE	CR	Factor intercorrelations	
					Factor 1: Exploring the patient perspective	Factor 2: Sharing information and power
Factor 1: Exploring the patient perspective	0.88	0.414	0.643	0.863	1	
Factor 2: Sharing information and power	0.90	0.422	0.650	0.877	0.71	1
Factor 3: Dealing with communicative challenges	0.71	0.346	0.588	0.718	0.53	0.54

*Note. n* = 258; AVE: average variance extracted; CR: composite reliability.

**Table 6.** Construct validity of the SEPCQ-24-GER.

	Sex^a^	Occupational group	Occupational experience (in years)
SEPCQ-24-GER, factor 1: Exploring the patient perspective	*t* = −1.275	*F* = 2.802	*r* = .130
	*p* = .203	***p* = .027**	***p* = .044**
	*d* = −0.163	*η*² = .043	
SEPCQ-24-GER, factor 2: Sharing information and power	*t* = 1.555	*F* = 6.517	*r* = .296
	*p* = .121	***p* < .001**	***p* < .001**
	*d* = 0.199	*η*² = .095	
SEPCQ-24-GER, factor 3: Dealing with communicative challenges	*t* = 2.575	*F* = 2.178	*r* = .144
	***p* = .011**	*p* = .072	***p* = .023**
	*d* = 0.329	*η*² = .034	
SEPCQ-24-GER	*t* = 0.886	*F* = 4.741	*r* = .240
	*p* = .376	***p* < .001**	***p* < .001**
	*d* = 0.113	*η*² = .072	

^a^ *Note. N* = 257, ‘Diverse/Non-binary’ has been excluded for this analysis.

## Discussion

The aim of the study was the translation and adaptation of the SEPCQ-27 into German as well as a preliminary evaluation of its psychometric properties in a sample of oncology physicians by exploring the factor structure and fit to the original and adapted models with the help of EFA and CFA as well as analyzing the internal consistency and convergent validity.

While the authors of the SEPCQ-27 found the items to be normally distributed with the suggested 5-point Likert scale (coded 0–4), the items are left-skewed in our sample with skewness values ranging from −1.177 to 0.052. Future analyses should verify this result and discuss if adjustments to the response format might be necessary.

Our analyses showed higher correlations between the factors than in the original SEPCQ-27 model [25]. Although no cross-loadings occurred, discriminant validity could be limited according to the Fornell Larcker criterion and needs further investigation. To the best of our authors’ knowledge, no reference values for discriminant validity have been reported until now. The items 2, 3, and 16 had low factor loadings (< 0.5) in EFA, which persisted in CFA for item 2 and 3 (data not shown). In future analyses, item 2 (‘Record a complete medical history’) and 3 (‘Accept when there is no longer curative treatment for the patient’) should be critically reviewed and could be candidates for item reductions.

Overall, EFA reproduced the original three-dimensional factor structure of the SEPCQ-27 with only three items (16, 19 and 24) loading on different factors. Omitting these three items in CFA led to the best model fit (*χ^2^/df* = 1.8, CFI = 0.92, TLI = 0.91, SRMR = 0.05, RMSEA = 0.06). Hence, the final model is a reduced three-dimensional construct with 24 items (SEPCQ-24-GER).

Possible reasons for the loading of items 16, 19 and 24 on the different factors could be that physicians in oncology in our German sample (i) rated a strong negative emotional reaction (angry) from patients as a challenge to explore the patient’s perspective rather than as a communicative challenge (item 16), (ii) rated professional disagreement (e.g. due to different treatment guidelines) as a challenge to share this information and the potential decision conflict with the patient rather than a communicative challenge (item 19), (iii) and rated a palliative situation as a communicative challenge rather than a challenge to explore the patient’s perspective (item 24). However, the low factor loadings of these three items led to their exclusion here, also because of minor thematic redundancies with other items (palliative situation, strong patient’s emotions).

The analysis of SEPCQ-24-GER internal consistency (Cronbach’s alpha) showed acceptable to good results with similar patterns of internal consistency for the original SEPCQ-27 model consisting of medical students and physicians [25]. The AVE failed to reach the defined threshold of 0.5 for all three dimensions. In case the AVE is below 0.5, convergent validity is still acceptable if CR > 0.6 according to Fornell and Larcker [43]. Since CR was > 0.7 for all three dimensions, convergent validity is assumed to be acceptable.

In terms of construct validity, the hypotheses that the SEPCQ-24-GER is positively correlated with longer professional experience and higher hierarchical level were confirmed. Sex (men/women) seems to play a rather minor role. This important aspect of validity should also be investigated in the future. In addition, the variables mentioned here should be considered as possible confounders in future multivariate analyses in which the SEPCQ-24-GER is used, e.g. as an outcome.

### Limitations and strengths

The presented findings should be considered in the light of methodological limitations. As the sample consists exclusively of physicians from oncology wards, the transfer and generalization to other specialized clinical areas remains to be studied. A classical test theory approach was followed rather than an item response theory approach, which would have the advantage of providing increased item level psychometric information. However, in order to be comparable to the original SEPCQ-27 version, we used classical test theory. The sample size did not allow for a split sample validation. Test-retest reliability was not tested because of the interventional approach of the original study. For future validation of the instrument, the use of a mean score rather than a sum score can be discussed, potentially facilitating comparability between studies. Future research should further explore the validity of the SEPCQ-24-GER, especially concerning the applicability by validations in different contexts, different and larger samples of physicians on a multi-institutional or national level, as well as criterion-related validity. Moreover, the sensitivity to change remains to be investigated. Compared to the original SEPCQ-27 [25], the items in our study were left-skewed. In the SEPCQ-27 version lower mean values are reported for medical students. Our data also show that the mean and median value of the SEPCQ-24-GER increases with work experience and differs between occupational groups. Therefore, the results in our sample appear to be comparable with the subsample of physicians tested with the original SEPCQ-27 version. A limited number of variables were employed in the testing of construct validity. Moreover, discriminant validity of the instrument should be investigated in the aforementioned manner (e.g. items 2 and 3).

The sample, on the one hand, includes physicians from every hierarchy level which exists in the German healthcare setting, on the other hand, physicians in the sample are comparatively young. Furthermore, physicians from oncological ward units where patients with nearly all oncological diseases are treated were included in the sample. Our findings are, therefore, likely to be widely generalizable to oncological healthcare settings with the SEPCQ-24-GER having the potential to be a valuable measure when assessing self-efficacy in patient-centeredness and patient-centered communication in oncology. In addition, the 24-item version of the instrument will also be validated for nurses in oncology in German which allows analyzing differences between these two groups.

### Implications for research and practice

A few studies have already explored associations between patient-centered care in oncology and outcomes for patients and physicians as well as the patient-physician relationship as perceived by patients [10,19]. The SEPCQ-24-GER provides a relevant additional instrument to the field with its focus on the perceived behavioral and skill-oriented aspects of patient-centeredness. The instrument captures the confidence of physicians in their ability to exert specific patient-centered behaviors, a focus which differs from existing German language instruments [24]. Thereby, the instrument can be used in multivariate effect measurements as (in-)dependent variable and potentially for evaluating interventions facilitating communications skills of physicians, patient-centeredness and patient-centered communication. Furthermore, our group is currently validating this instrument in German for nurses in oncology, which will allow evaluating oncology nurse-led interventions, interprofessional team communication skills trainings or differences between these two groups in the future. This could help elucidate the question whether self-efficacy in patient-centeredness is a more generic concept or is more related to specific skills and behaviors represented by the individual items. Future research by our group will concentrate on this important aspect of interprofessional healthcare practice in oncology [36].

## Conclusions

With the SEPCQ-24-GER, a theory-based instrument for the measurement of self-efficacy in patient-centeredness among physicians in oncology is now available in German (see Appendix, Table A1), and preliminary evidence for the reliability and validity aspects presented here can be stated. Future research should focus on further measures of reliability, sensitivity to change and validation within different samples. It can be used as an instrument in studies of patient-centered communication and communication skills trainings for physicians. As the instrument will also be adapted for nurses in oncology, further comparative analyses will be carried out and practice implications discussed.

## Data Availability

Data for this study are kept at the Center for Health Communication and Health Services Research, University Hospital Bonn, University of Bonn, Germany. The datasets generated and analyzed during the current study are not publicly available due to terms of written informed consent to which the participants agreed but are available from the corresponding author on reasonable request.

## Appendix A

**Table A1.** German version of the SEPCQ-27 and SEPCQ-24-GER.

	SEPCQ-24 German version (SEPCQ-24-GER)
Introduction	Im Folgenden werden anhand mehrerer Aussagen Aspekte beschrieben, die sich auf Ihre Fähigkeit beziehen, mit Patienten zu kommunizieren. Bitte lesen Sie jede Aussage sorgfältig. Beurteilen Sie anschließend Ihre Fähigkeit, auf die beschriebene Weise mit Patienten zu kommunizieren. Beantworten Sie alle Fragen und bewerten Sie so gut wie möglich, inwieweit Sie in der Lage sind, sich wie beschrieben zu verhalten. Bitte beantworten Sie alle Fragen so ehrlich wie möglich. Es geht nicht darum, in welchem Maße Sie dieses Verhalten gerne zeigen würden, sondern um Ihre realistischen Einschätzungen. Ich bin davon überzeugt, dass ich in der Lage bin…
Item 1	dem Patienten das Gefühl zu vermitteln, dass ich ehrlich daran interessiert bin, was er über seine Situation denkt.
Item 2	eine umfassende Anamnese zu erheben.
Item 3	zu akzeptieren, dass es keine weitere kurative Behandlung mehr für den Patienten gibt.
Item 4	dem Patienten das Gefühl zu geben, dass ich Zeit habe zuzuhören.
Item 5	die Gedanken und Gefühle des Patienten anzuerkennen.
Item 6	eine Einigung über den Behandlungsplan mit dem Patienten zu erzielen.
Item 7	den Patienten bei Entscheidungen zur Behandlung zu beraten und zu unterstützen.
Item 8	wahrzunehmen, wenn meine Gefühle die Kommunikation mit dem Patienten beeinflussen.
Item 9	aufmerksam zu sein und auf die Bedürfnisse des Patienten einzugehen.
Item 10	zu erkennen, wenn der Patient ängstlich oder besorgt ist.
Item 11	sicherzustellen, dass der Patient ausreichend informiert ist, eine Entscheidung zu treffen.
Item 12	dem Patienten die Diagnose und den Behandlungsplan verständlich zu erklären.
Item 13	mit meinen eigenen Emotionen in schwierigen Situationen umzugehen.
Item 14	den Patienten fürsorglich zu behandeln.
Item 15	Dinge so zu erklären, dass sich der Patient gut informiert fühlt.
Item 16*	die Beziehung zum Patienten aufrechtzuerhalten, wenn er wütend ist.
Item 17	dem Patienten empathisch zu begegnen.
Item 18	den Patienten über die erwarteten Nebenwirkungen so aufzuklären, dass er sie versteht.
Item 19*	bei fachlicher Uneinigkeit über die Diagnose und Behandlung darauf fokussiert zu bleiben, was das Beste für den Patienten ist.
Item 20	dem Patienten das Gefühl zu geben, dass er über vertrauliche Angelegenheiten mit mir sprechen kann.
Item 21	zu erklären, wie die Behandlung abläuft bzw. ablaufen sollte.
Item 22	mich nicht von einer Voreingenommenheit gegenüber dem Patienten beeinflussen zu lassen.
Item 23	aufrichtiges Interesse an dem Patienten und seiner Situation zu zeigen.
Item 24*	den Schwerpunkt auf Anteilnahme, Fürsorge und symptomatische Behandlung zu legen, wenn es keine kurative Behandlung gibt.
Item 25	dem Patienten verständlich zu erklären, wie sich die Behandlung wahrscheinlich auf seinen Zustand auswirkt.
Item 26	die Behandlungsverfahren so zu erklären, dass der Patient sie versteht.
Item 27	meine persönlichen Ansichten von meiner professionellen Herangehensweise zu trennen.
Scale	in sehr geringem Maß – in sehr hohem Maß (5-point Likert scale)

*Note**. The items 16, 19 and 24 were omitted resulting in the final SEPCQ-24 German version (SEPCQ-24-GER).

## References

[1] Scholl I, Zill JM, Härter M, et al. An integrative model of patient-centeredness - a systematic review and concept analysis. PLoS One. 2014;9(9):e107828. doi: 10.1371/journal.pone.0107828. 25229640 PMC4168256

[2] Stewart M. Patient-centered medicine - transforming the clinical method. 2nd ed. Abingdon (UK): Radcliffe Medical Press; 2003.

[3] Stiefel F, Bourquin C, Salmon P, et al. Communication and support of patients and caregivers in chronic cancer care: ESMO Clinical Practice Guideline. ESMO Open. 2024;9(7):103496. doi: 10.1016/j.esmoop.2024.103496 39089769. 39089769 PMC11360426

[4] Varpio L, Paradis E, Uijtdehaage S, et al. The distinctions between theory, theoretical framework, and conceptual framework. Acad Med. 2020;95(7):989–994. doi: 10.1097/ACM.0000000000003075. 31725464

[5] McCormack LA, Treiman K, Rupert D, et al. Measuring patient-centered communication in cancer care: a literature review and the development of a systematic approach. Soc Sci Med. 2011;72(7):1085–1095. doi: 10.1016/j.socscimed.2011.01.020. 21376443

[6] Finset A. Research on person-centred clinical care. J Eval Clin Pract. 2011;17(2):384–386. doi: 10.1111/j.1365-2753.2010.01608.x. 21208349

[7] Venetis MK, Robinson JD, Turkiewicz KL, et al. An evidence base for patient-centered cancer care: a meta-analysis of studies of observed communication between cancer specialists and their patients. Patient Educ Couns. 2009;77(3):379–383. doi: 10.1016/j.pec.2009.09.015. 19836920

[8] Henry SG, Fuhrel-Forbis A, Rogers MAM, et al. Association between nonverbal communication during clinical interactions and outcomes: a systematic review and meta-analysis. Patient Educ Couns. 2012;86(3):297–315. doi: 10.1016/j.pec.2011.07.006. 21824738

[9] Oliveira VC, Refshauge KM, Ferreira ML, et al. Communication that values patient autonomy is associated with satisfaction with care: a systematic review. J Physiother. 2012;58(4):215–229. doi: 10.1016/S1836-9553(12)70123-6. 23177224

[10] Stewart M. Effective physician-patient communication and health outcomes - a review. Can Med Assoc J. 1995;152:1423–1433. 7728691 PMC1337906

[11] Griffin SJ, Kinmonth A-L, Veltman MWM, et al. Effect on health-related outcomes of interventions to alter the interaction between patients and practitioners: a systematic review of trials. Ann Fam Med. 2004;2(6):595–608. doi: 10.1370/afm.142. 15576546 PMC1466743

[12] Ratanawongsa N, Roter D, Beach MC, et al. Physician burnout and patient-physician communication during primary care encounters. J Gen Intern Med. 2008;23(10):1581–1588. doi: 10.1007/s11606-008-0702-1. 18618195 PMC2533387

[13] Passalacqua SA, Segrin C. The effect of resident physician stress, burnout, and empathy on patient-centered communication during the long-call shift. Health Commun. 2012;27(5):449–456. doi: 10.1080/10410236.2011.606527. 21970629

[14] Krasner MS, Epstein RM, Beckman H, et al. Association of an educational program in mindful communication with burnout, empathy, and attitudes among primary care physicians. JAMA. 2009;302(12):1284–1293. doi: 10.1001/jama.2009.1384. 19773563

[15] Shanafelt TD. Enhancing meaning in work: a prescription for preventing physician burnout and promoting patient-centered care. JAMA. 2009;302(12):1338–1340. doi: 10.1001/jama.2009.1385. 19773573

[16] Kissane DW, Bylund CL, Banerjee SC, et al. Communication skills training for oncology professionals. J Clin Oncol. 2012;30(11):1242–1247. doi: 10.1200/JCO.2011.39.6184. 22412145 PMC3341141

[17] Moore PM, Rivera MS, Grez AM, et al. Communication skills training for healthcare professionals working with people who have cancer. Cochrane Database Syst Rev. 2013;2013(3):CD003751. Cochrane Database Syst Rev. 2013;3. doi: 10.1002/14651858.CD003751.pub3. 23543521 PMC6457800

[18] Barth J, Lannen P. Efficacy of communication skills training courses in oncology: a systematic review and meta-analysis. Ann Oncol. 2011;22(5):1030–1040. doi: 10.1093/annonc/mdq441. 20974653

[19] Uitterhoeve RJ, Bensing JM, Grol RP, et al. The effect of communication skills training on patient outcomes in cancer care: a systematic review of the literature. Eur J Cancer Care (Engl). 2010;19(4):442–457. doi: 10.1111/j.1365-2354.2009.01082.x. 20030702

[20] Berkhof M, van Rijssen HJ, Schellart AJM, et al. Effective training strategies for teaching communication skills to physicians: an overview of systematic reviews. Patient Educ Couns. 2011;84(2):152–162. doi: 10.1016/j.pec.2010.06.010. 20673620

[21] Selman LE, Brighton LJ, Hawkins A, et al. The effect of communication skills training for generalist palliative care providers on patient-reported outcomes and clinician behaviors: a systematic review and meta-analysis. J Pain Symptom Manage. 2017;54(3):404–416.e5. doi: 10.1016/j.jpainsymman.2017.04.007. 28778560

[22] Vitinius F, Sonntag B, Barthel Y, et al. KoMPASS - Konzeption, Implementierung und Erfahrungen mit einem strukturierten Kommunikationstraining für onkologisch tätige Ärzte. [KoMPASS–design, implementation and experiences concerning a structured communication skills training for physicians dealing with oncology]. Psychother Psychosom Med Psychol. 2013;63(12):482–488. doi: 10.1055/s-0033-1341468. 23677627

[23] Karger A, Geiser F, Vitinius F, et al. Communication skills trainings: subjective appraisal of physicians from five cancer centres in North Rhine, Germany. Oncol Res Treat. 2017;40(9):496–501. doi: 10.1159/000479113. 28750399

[24] Zill JM, Christalle E, Müller E, et al. Measurement of physician-patient communication–a systematic review. PLoS One. 2014;9(12):e112637. doi: 10.1371/journal.pone.0112637. 25532118 PMC4273948

[25] Zachariae R, O’Connor M, Lassesen B, et al. The self-efficacy in patient-centeredness questionnaire - a new measure of medical student and physician confidence in exhibiting patient-centered behaviors. BMC Med Educ. 2015;15(1):150. doi: 10.1186/s12909-015-0427-x. 26374729 PMC4572680

[26] Wünsch A, Bergelt C, Götze H, et al. Kommunikationstrainings für onkologisch tätige Ärzt*innen in Deutschland. Forum. 2021;36(5):391–395. doi: 10.1007/s12312-021-00972-7.

[27] Wünsch A, Boden MJ, Pärschke PP, et al. Com-On Questionnaire: development and validation of a questionnaire for evaluating communication skills of oncologists. Eur J Cancer Care (Engl). 2022;31(6):e13684. doi: 10.1111/ecc.13684. 35985987

[28] Stensrud TL, Mjaaland TA, Finset A. Communication and mental health in general practice: physicians’ self-perceived learning needs and self-efficacy. Ment Health Fam Med. 2012;9(3):201–209. 23997826 PMC3622912

[29] Bandura A. Self-efficacy - the exercise of control. New York: Freeman and Company; 1997.

[30] Bandura A. Self-efficacy: toward a unifying theory of behavioral change. Psychol Rev. 1977;84(2):191–215. doi: 10.1037/0033-295X.84.2.191. 847061

[31] Parle M, Maguire P, Heaven C. The development of a training model to improve health professionals’ skills, self-efficacy and outcome expectancies when communicating with cancer patients. Soc Sci Med. 1997;44(2):231–240. doi: 10.1016/S0277-9536(96)00148-7. 9015875

[32] Stiefel F, Favre N, Despland JN. Communication skills training in oncology: it works!. Recent Results Cancer Res. 2006;168:113–119. doi: 10.1007/3-540-30758-3_11. 17073197

[33] Dwamena F, Holmes-Rovner M, Gaulden CM, et al. Interventions for providers to promote a patient-centred approach in clinical consultations. Cochrane Database Syst Rev. 2012;12(12):CD003267. doi: 10.1002/14651858.CD003267.pub2. 23235595 PMC9947219

[34] Pedersen K, Bennedsen A, Rungø B, et al. Evaluating the effectiveness of video cases to improve patient-centeredness in psychiatry: a quasi-experimental study. Int J Med Educ. 2019;10:195–202. doi: 10.5116/ijme.5d9b.1e88. 31658442 PMC7246115

[35] Mokkink LB, Terwee CB, Patrick DL, et al. The COSMIN checklist for assessing the methodological quality of studies on measurement properties of health status measurement instruments: an international Delphi study. Qual Life Res. 2010;19(4):539–549. doi: 10.1007/s11136-010-9606-8. 20169472 PMC2852520

[36] Karger A, Petermann-Meyer A, Vitinius F, et al. Effectiveness of interprofessional communication skills training for oncology teams: study protocol for a three-arm cluster randomised trial (KommRhein Interpro). BMJ Open. 2022;12(12):e062073. doi: 10.1136/bmjopen-2022-062073. PMC9806046 36581438

[37] Dillman DA. Mail and telephone surveys: the total design method. New York: Wiley-Interscience; 1978.

[38] Guillemin F, Bombardier C, Beaton D. Cross-cultural adaptation of health-related quality of life measures: literature review and proposed guidelines. J Clin Epidemiol. 1993;46(12):1417–1432. doi: 10.1016/0895-4356(93)90142-n. 8263569

[39] van Someren MW, Barnard YF, Sandberg JA. The think aloud method: A practical guide to modelling cognitive processes. London Academic Press; 1994.

[40] Wirtz M. Uber das Problem fehlender Werte: Wie der Einfluss fehlender Informationen auf Analyseergebnisse entdeckt und reduziert werden kann. [On the problem of missing data: how to identify and reduce the impact of missing data on findings of data analysis]. Rehabilitation (Stuttg). 2004;43(2):109–115. doi: 10.1055/s-2003-814839. 15100920

[41] Field A. Discovering statistics using SPSS: (and sex and drugs and rock ‘n’ roll). 3rd ed. Los Angeles: Sage Publications; 2009.

[42] Hair JF, Babin BJ, Anderson RE, et al. Multivariate Data Analysis: Pearson New International Edition. 7th ed. Harlow: Pearson Education Limited; 2013.

[43] Fornell C, Larcker DF. Evaluating structural equation models with unobservable variables and measurement error. Journal of Marketing Research. 1981;18(1):39–50. doi: 10.2307/3151312.

